# CarFreeMe™-Dementia: Potential Benefits of a Driving Retirement Intervention Supporting Persons With Dementia and Their Families

**DOI:** 10.1093/geroni/igae022

**Published:** 2024-03-01

**Authors:** Colleen M Peterson, Stephanie Ingvalson, Robyn W Birkeland, Katie W Louwagie, Theresa L Scott, Nancy A Pachana, Jacki Liddle, Louise Gustafsson, Joseph E Gaugler

**Affiliations:** University of Michigan Transportation Research Institute, University of Michigan, Ann Arbor, Michigan, USA; School of Public Health, University of Minnesota, Minneapolis, Minnesota, USA; School of Public Health, University of Minnesota, Minneapolis, Minnesota, USA; School of Public Health, University of Minnesota, Minneapolis, Minnesota, USA; School of Psychology, University of Queensland, Brisbane, Queensland, Australia; School of Psychology, University of Queensland, Brisbane, Queensland, Australia; School of Health and Rehabilitation Sciences, University of Queensland, Brisbane, Queensland, Australia; Department of Occupational Therapy, Princess Alexandra Hospital, Brisbane, Queensland, Australia; School of Health Sciences and Social Work, Griffith University, Nathan, Queensland, Australia; School of Public Health, University of Minnesota, Minneapolis, Minnesota, USA

**Keywords:** Caregiver stress, Dementia, Driving, Evaluation, Qualitative analysis, Thematic analysis

## Abstract

**Background and Objectives:**

Driving retirement can be a necessary but challenging and emotionally complex transition, especially for people living with dementia. This pilot study evaluated the utility of CarFreeMe™-Dementia (CFM™-D), a telehealth intervention providing tailored education and social support to those living with dementia and their care partners, as they prepare for or adjust to driving retirement. Delivered by empathetic health professionals, CFM™-D is a person-centric, flexible program tailored to address challenges specific to the participants’ driving retirement stage and individualized contexts.

**Research Design and Methods:**

A single-arm, mixed-methods design was used to follow participants over a 6-month period. Participants received CFM™-D, a 7–8-module semistructured intervention, including education and planning support for driving retirement (impact of dementia, transportation options) and emotional adjustment (grief and loss, stress management). Surveys evaluated the perceived utility of intervention components as well as changes in well-being and readiness for driving retirement over time. An open-ended survey item and semistructured interviews provided additional feedback and a contextual understanding of the empirical data.

**Results:**

A total of 50 families enrolled (17 care partners, 16 retiring/retired drivers with memory loss, and 17 care partner-retiring/retired driver dyads). Nearly all participants would recommend the intervention. Care partners reported significantly reduced (*p* < .05) isolation and relationship strain, and retiring drivers reported significant reductions in depressive symptoms. Driving retirement preparedness scores improved. Driving retirement phase, enrolling as a dyad, and retiring driver cognitive/functional impairment were associated with these outcomes. Participants also engaged in more driving retirement activities outside of the intervention (e.g., talking with health professionals).

**Discussion and Implications:**

CFM™-D is a useful intervention for retiring drivers with dementia and their family members, with preliminary data suggesting it supports improved well-being and driving retirement preparedness. A randomized controlled trial is needed to determine the efficacy of the CFM™-D intervention and future translation needs.

**Translational Significance:** There are currently no evidence-based care-provider interventions to facilitate driving retirement with persons living with dementia and their families. Interventions that do exist are mainly limited to web-based self-guided tools. CarFreeMe™-Dementia fills a critical care gap with its flexible and tailored coaching approach that empowers the person with dementia to engage in driving retirement decisions and attend to a family’s emotional and practical needs before, during, and after driving retirement. Pilot findings show initial support for the CarFreeMe™-Dementia intervention, suggesting it may be an effective clinical or community tool addressing practical and emotional aspects of dementia and driving retirement.

In 2021, there were over 227 million licensed drivers in the United States, among the highest number of drivers per capita in the world (OECD, 2023). The number of licensed drivers in the United States 65 years and older has been steadily increasing as our population ages (Mizenko et al., 2014; Naumann et al., 2014). Driving is the principal form of transportation for most older adults, providing access to services and social activities (O’Neill, 2015). Thus, retirement from driving is a major life transition and is often experienced as a loss of control, independence, competence, autonomy, and social engagement (Pachana et al., 2017; Sanford et al., 2019). Driving retirement also can result in the loss of community connectivity and social participation. For people with dementia, this can lead to a more rapid decline in cognition (De Silva et al., 2019) and have a negative impact on health and wellbeing (Chihuri et al., 2016).

Although a diagnosis of dementia does not necessitate immediate driving cessation (Man-Son-Hing et al., 2007), the progressive and degenerative effects of dementia can place the driver, and consequently, other road users, at risk and heighten the need for driving termination. Driving is a complex task that is compromised by various dementia-related deficits, such as impairments in visuospatial skills, situational awareness, judgment and decision-making, psychomotor functioning, reaction time, and wayfinding (Ott & Daiello, 2010). Furthermore, dementia can impair an individual’s capacity to self-monitor their limitations and driving safety (Pachana & Petriwskyj, 2006; Scott et al., 2020a), increasing the risk of crashes (Man-Son-Hing et al., 2007).

Research has shown that care partners and family members can play a critical role in the individual’s decision to stop driving (Johnson, 2008). However, conversations around driving retirement are often emotionally charged and sometimes avoided until a crisis occurs (Liddle et al., 2013). Unsurprisingly, driving retirement has been described as one of the most challenging issues faced by people living with dementia and their physicians, care partners, and other family members (Liddle et al., 2016; Scott et al., 2020a).

Without practical and emotional support like access to alternative transport or grief counseling, driving retirement can be a potentially traumatic experience for people with dementia (Holden & Pusey, 2021). Support for retiring/retired drivers (collectively referred to as retiring drivers) can help people with dementia transition to alternative modes of transportation; promote their safety and the safety of other road users; as well as maintain their sense of agency, independence, and social participation.

## Interventions to Support Driving Retirement

Despite the concerns for safety and impact of driving retirement on quality of life for people with dementia and their care partners, service gaps are evident. There are currently no empirically tested care-provider-delivered interventions to facilitate driving retirement for people with dementia available in routine clinical practice (Holden & Pusey, 2021). Stasiulis and colleagues (2023) recently profiled a range of tools designed to support driving retirement and created the Driving and Dementia Roadmap repository. Most interventions are self-guided resources (e.g., Byszewski et al., 2017; Jouk & Tuokko, 2017) or decision-making tools (e.g., Carmody et al., 2015; Chang et al., 2021; Polzer et al., 2020). They specifically noted a lack of tools that target the emotional aspects of driving retirement, which is critical to initiating and adjusting to driving retirement (Musselwhite, 2023). Consistent limitations noted throughout driving retirement research include the need for information about local transportation options, legal and licensing issues (Holden & Pusey, 2021; Liddle et al., 2013), the changing needs during the driving retirement process, and the impact of driving retirement on relationships (Liddle et al., 2013, 2016).

## The CarFreeMe-Dementia Intervention

The CarFreeMe program of research (formerly UQDRIVE) managed these deficits by creating an intervention that: (a) is delivered by health professionals who could elicit participant needs and adapt the program according to their context, (b) attends to challenges at each stage of driving retirement (planning for, actively implementing, or adjusting to), (c) targets participant-specific goals, and (d) provides local information (Liddle et al., 2007). This intervention was adapted for people living with dementia, CarFreeMe™-Dementia (CFM™-D), filling a gap in existing interventions by including both the person with dementia and their care partner(s) in planning and decision-making throughout each phase of the driving retirement process (Scott et al., 2020b). The CFM™-D intervention provides person-centered, flexible, and individually tailored education and social support, with a goal of improving outcomes such as community mobility, personal well-being, and driving retirement preparedness (Peterson et al., 2023; Scott et al., 2019; 2020b). The present study describes Phase II of a two-phase study evaluating the CFM™-D intervention modified for a U.S. audience. A primarily qualitative analysis of 16 care partner/retiring driver dyads’ experiences in Phase I showed acceptability and feasibility of the CFM™-D intervention and identified initial themes for mechanisms of benefit (Peterson et al., 2023).

The CFM™-D intervention was delivered by two study coaches to retiring drivers with dementia and their care partners, individually or as a dyad, via phone or video conferencing (i.e., Zoom). The two study coaches (with a PhD in clinical psychology and a Doctor of Nursing Practice), both of whom are considerably experienced working with this population in intervention research, were trained and certified by the CFM™-D developer. Specifically, the semistructured CFM™-D intervention involved an initial interview with the coach for tailoring purposes, followed by seven coach-led psychosocial and psychoeducational content modules. The coaches guided participants through modules on dementia education and associated changes that affect driving, balancing independence and safety, adjusting to loss, coping strategies, experiences from retired drivers, lifestyle planning, advocacy, problem-solving, and alternative transportation options including local transit services. An 8^th^ module for the care partner(s) alone, if applicable, covered self-care and driving retirement conversation tips. Developmental work indicated that all aspects of the intervention were required to address the often-evolving range of driving cessation needs and changes (Gustafsson et al., 2011).

The intervention module content order and topics emphasized were tailored to meet participant needs (e.g., phase of driving retirement, participant-identified priorities, and immediate concerns). Module content was reviewed in four (minimally) to eight, typically 1-h intervention sessions and took place on an approximately weekly basis during the 3 months following baseline survey completion. The coaches were empathetic and the sessions were conversational. Coaches were also available for ad hoc communication during and following intervention completion to provide participants with prompt and responsive support outside of sessions. Participants were offered a companion workbook (digital or hardcopy) with module content and related resources for them to follow along in the sessions and reference later. Based on Phase I feedback (Peterson et al., 2023), changes to the CFM™-D for Phase II included highlighting a range of alternatives to driving and removal of duplicate content. See Table 1 for a more detailed outline of intervention module details and further information in Liddle et al. (2007, 2013); Peterson et al. (2023); Scott et al. (2019); and Scott et al. (2020b).

**Table 1.** CarFreeMe™-Dementia Intervention Module Descriptions

Module Topic	Description
Module 1: Living with dementia	Focuses on the changes that may occur with dementia and strategies to live positively.
Module 2: Balancing independence and safety	Highlights driving safety and impact of dementia on necessary driving skills.
Module 3: Adjusting to losses and changes	Covers expected changes and strategies to adjust to lifestyle and feelings of loss and grief.
Module 4: Experience of retiring from driving	Covers what it is like to give up driving; Stories that show different ways to adjust are shared.
Module 5: Alternative transport	Covers a range of driving alternatives and where to learn more.
Module 6: Lifestyle planning	Covers issues to consider in planning for achieving a balanced lifestyle.
Module 7: Advocacy and support	Focuses on available services and steps to take to make service providers aware of needs.
Module 8: Care partner only	Focuses on self-care and coping strategies; Shares tips for talking about driving cessation with the person with AD/ADRD.
Ad hoc	Explores ongoing concerns and reviews relevant topics

*Notes*: AD = Alzheimer’s disease; ADRD = Alzheimer’s disease and related dementias.

The primary aim of the present Phase II study was to evaluate the utility of the intervention to affect the well-being and driving retirement preparedness of people with dementia and their care partners in the United States. The Phase II study was adequately powered to detect changes in these measures to demonstrate preliminary efficacy as well as empirically delineate contextual mechanisms of benefit. Given the importance of social support (Holden & Pusey, 2021; Liddle et al., 2023) and different driving retirement stage challenges (Liddle et al., 2013; Peterson et al., 2023), we hypothesized that dyad status and whether the person with dementia was preparing for or adjusting to driving retirement would have significant effects on intervention receipt, well-being, and driving retirement preparedness.

## Method

### Participants

The study was approved by the University of Minnesota Institutional Review Board [STUDY00009343]. Recruitment efforts involved greater outreach to enroll care partners and persons in earlier stages of dementia. Efforts included utilization of caregiver registry email lists maintained by the University of Minnesota, as well as Banner Alzheimer’s Institute, an external recruitment agency that sent targeted emails to potential participants in their Alzheimer’s Prevention Registry. Other recruitment efforts included partnerships with memory care clinics, informational flyers shared at educational outreach events and with community organizations, the University of Minnesota’s study website, and word of mouth.

Research staff reviewed project details and completed a screening questionnaire to determine eligibility of interested retiring drivers and/or their care partners. Eligible retiring drivers were those that: (a) had either been diagnosed with Alzheimer’s disease or related dementias (ADRD) or they or their care partner endorsed concerns about their memory or cognition and: (b) were planning for or actively implementing driving retirement or had already retired from driving (adjusting to driving retirement). Eligible care partners were family members who provided unpaid support to the retiring driver. Additionally, eligible participants were: (a) at least 21 years old; (b) English-speaking, and (c) residing in the United States. Individuals were ineligible if they resided in a nursing home, initiated or changed a psychotropic medication within the past 3 months, or were not receiving treatment for a mental health condition that worsened in the past 6 months.

Fifty individuals or retiring driver/care partner dyads provided informed consent and enrolled in the study between September 2021 and May 2022: 16 retiring drivers, 17 care partners, and 17 retiring driver/care partner dyads. Most dyads consisted of spousal pairs (16 of 17 dyads). See Supplementary Figure 1 for the participant flow.

### Procedures

Quantitative survey data was collected from participants at baseline, 3 months, and 6 months via mail, telephone, or electronically via Qualtrics. Additional intervention feedback was collected in qualitative form from an open-ended survey question and semistructured interviews conducted by the study coordinator. Thirty participants were purposely selected for interviews based on geographical factors (rural vs suburban), general cognition (e.g., anticipated ability to provide feedback), dyad enrollment, and phase of driving retirement. Retiring drivers completed interviews following the 3-month surveys to facilitate better recall of the intervention. Care partners completed their interview after the 6-month survey. Interviews were transcribed for analysis.

### Measures

Measures in the present study were revised and refined based on Phase I analyses and other feedback to better capture CFM™-D mechanisms of benefit and moderators. Demographic information on care partners and retiring drivers was collected at baseline (e.g., gender and living arrangement). In addition, each timepoint used validated measures to assess retiring drivers’ cognitive impairment (Pearlin et al., 1990; Teri et al., 1992); functional ability (Katz et al., 1963), community service use (Sonnega et al., 2017); and well-being, including caregiver strain (Bass et al., 1996), loneliness (De Jong Gierveld & Van Tilburg, 2006, 2010), relationship closeness (Whitlatch et al., 2001), and depressive symptoms (Yesavage & Sheikh, 1986). Driving-related data included driving phase status, readiness for mobility transition (Meuser et al., 2013), safe transport (Iverson et al., 2010), confidence in driving retirement (Scott et al., 2019), and driving retirement-related activities (e.g., restricted driving, talking with family about retirement). Follow-up surveys (3- and 6-months) also included an evaluation of treatment receipt and delivery. Care partners enrolling with a retiring driver were asked to complete some measures on their behalf. See Supplementary Table 1 for measure and distribution details.

Qualitative data were derived from the treatment receipt measure and the interview. The treatment receipt measure included one open-ended item that read “Please add any other ways that this experience has been helpful to you, or any other comments that you have about CarFreeMe.” Example interview questions include: “What did you get out of the CarFreeMe program,” “What did you feel was not helpful or was missing from the program,” and “Did the program make it easier to talk about driving issues?” Supplementary Text 1 has the full semistructured interview guide.

## Analysis

We described demographic, well-being, and driving-related characteristics of the participants at baseline, 3-, and 6-months. We conducted paired *t* tests and Chi-square analyses as appropriate between baseline and follow-up. To examine correlates with change over time for continuous outcomes, we ran mixed effect models using restricted maximum likelihood with identity covariance structures and accounted for small samples by specifying the Kenward–Roger method (Kenward & Roger, 1997). We used forward stepwise selection and standard model fit criteria (i.e., Akaike information criterion and Bayesian information criterion) to choose final models. Analyses were run in Stata17.

The current study was a concurrent partially mixed method design (Leech & Onwuegbuzie, 2009). Authors C.M.P., R.W.B., K.W.L., and S.I. independently reviewed a subset of the data from the interviews and C.M.P. reviewed the open-ended question responses from CFM™-D checklist. They discussed commonalities and specific examples to derive overall themes and supporting codes. For this study, we present quotes converging with the quantitative outcomes to offer a more contextual understanding of the findings.

## Results

### Participant Characteristics

The majority of care partners were women (88%), spouses of the retiring driver (74%), and had a mean age of 67 years old. Half of the enrolled retiring drivers were women (*n* = 16), averaging 73 years of age. Care partners who enrolled on their own were less likely to be the spouse of or live with, the retiring driver. Additionally, their retiring drivers (combining enrolled and not) had greater cognitive impairment but were not more likely to have a formal dementia diagnosis. Of enrolled retiring drivers, retiring drivers who enrolled without a care partner were more likely to be women and less likely to be White or indicate living with a care partner. Individual retiring drivers were also less likely to be formally diagnosed with ADRD and had better physical functioning, but greater memory impairment than dyadic retiring drivers. Table 2 shows further demographic details. See Supplementary Table 2 for statistical comparisons.

**Table 2.** Baseline Sample Characteristics

Characteristic	*n*	%	M	*SD*
Care partner (*n* = 34)				
Female	30	88.2		
Age			66.7	11.9
White	32	94.1		
Married	31	91.2		
Bachelor’s degree or higher	25	73.5		
Number of living children			1.9	1.5
Annual Income of US$80,000 or more^a^	16	47.1		
Spouse of care recipient	25	73.5		
Employed	6	17.6		
Enrolled retiring driver (*n* = 32)				
Female^*^	16	50.0		
Age			73.2	6.1
White^*^	28	87.5		
Married^*^	21	65.6		
Bachelor’s degree or higher	21	65.6		
Number of living children			2.0	1.4
Annual Income of US$30,000 or more^a^	24	77.4		
Lives with caregiver^*^	19	59.4		
Retiring Driver Dementia Characteristic				
Years recognizing memory concerns			5.1	5.6
Limitations to activities of daily living sum^*^			5.1	5.0
Memory impairment sum^*^			9.2	4.4
Dementia diagnosis^b^	11	34.4		
Retiring driver driving behaviors				
In last 3 years any…				
Traffic violation^*^	4	12.9		
Caused or in accident	6	19.4		
Accidents at fault	6	19.4		
Weekly miles driven				
0	12	38.7		
1–25	12	38.7		
26–50	6	19.4		
51–100	1	3.2		

*Notes*: M = mean; *SD* = Standard deviation.

^a^Median reported income on categorical scale.

^b^Does not include mild cognitive impairment.

^*^ *p* < .05 between retiring driver-dyad and retiring driver-individually enrolled.

### CarFreeMe™-Dementia Receipt

Participants completed an average of 6.7 sessions (range 1–13 sessions; *SD* = 2.20). The average session duration was 1 h (range 5–120 min; *SD* = 13.19). Overall, receipt of the CFM™-D was very positive (See Supplementary Table 3 for items). Qualitative data supported these findings. Approximately 96% of care partners agreed that they would recommend CFM™-D to others in similar situations to themselves at the 3-month follow-up, dropping slightly to about 92% at 6 months. ID60 (care partner; individual enrollee; and daughter) shared that her family “learned so much during the study about the progression of dementia and memory loss, and ways for loved ones to cope with the changes. We so appreciate the study and would definitely recommend it to others!” Approximately 91% of care partners agreed that CFM™-D helped them learn the importance of planning early for driving retirement. For example, ID180 (care partner; individual enrollee) shared, “I’m more prepared to look into things before [driving retirement is necessary] and arrange something at least with neighbors or something [so] that we can get things done.”

At 6 months, approximately 89% of retiring drivers agreed that CFM™-D helped them participate in their communities. As ID231 (retiring driver; individual enrollee) noted, “I’m even using some substitute transportation to a group that I go to. I’m actually taking this friend who can’t drive, but instead of my driving, we’re taking [a shared paratransit service].” Eighty-six percent of retiring drivers agreed that CFM™-D helped them to plan for driving retirement. ID361 (retiring driver; individual enrollee) shared:

[It] encouraged me to talk more to my friends here because there’s certain circumstances where they regret and/or resent having their car taken from them. And that kind of puts me in a position that I don’t want my family to [be in]. And made me aware of what I need to know and recognize when the time comes.

ID141 (retiring driver; individual enrollee) expressed “[CFM™-D] helped me codify my own experience … compare [it to what] other people have confronted … the nuances, the individual adaptations that are necessary or possible.”

Of note, retiring drivers indicated higher levels of agreement on several items at 3 months versus 6 months, possibly due to memory loss or increasing needs. For example, 96% of retiring drivers agreed that CFM™-D helped them to express their feelings about not driving anymore at 3 months, but 81% agreed at 6 months. Retiring drivers who had not stopped driving during the study were more likely to agree CFM™-D helped them to plan for when they will no longer drive (item #1; stopped driving at 3 months: χ^2^(1) = 7.6, *p* = .006; 6 months: χ^2^(1) = 4.2, *p* = .040). Overall, nearly all retiring drivers (~92%) said they would recommend the intervention to others.

#### Dyad status

The only differences found for care partners participating in the study individually versus as a member of a dyad were that dyadic care partners were much more likely to say the intervention helped them express concerns and feel validated (93.8% vs 50.0% agree; item #9) and to feel supported through the emotional adjustment of driving loss (93.8% vs 55.6% agree; item #10). As ID 260 (care partner; dyadic enrollee) expressed:

CarFreeMe helped my husband and me communicate more openly about his driving. I am more aware of my responsibility for intervening in unsafe driving as it emerges. The best part of the program was the support and encouragement it gave me through the individualized counseling on coping with my husband’s dementia. My husband also felt validated and supported.

For retiring drivers, treatment receipt by dyad status found just one difference: 100% of individual retiring drivers agreed the intervention helped them “Plan for when I will not drive anymore,” compared to 66.7% and 70.0% agreement reported by dyadic retiring drivers at 3- and 6-month follow-up, respectively. ID461 (retiring driver; individual enrollee) described how CFM™-D “[helped] me to narrow down what it is I specifically have to do to accomplish not driving, because I know that day is coming, so … I’m preparing myself so that it’s less stressful.”

#### Retention

Notably, 28 of 32 care partners completed all intervention sessions. However, a total of nine care partners were lost during later follow-up, seven of whom were individual enrollees. Attrition analyses of their and their retiring driver’s baseline demographics, well-being, and phase of driving retirement found a single difference: care partners lost to follow-up had fewer children (mean diff.(*SD*) = 1.09(0.57), *p* = .0326). See Supplementary Figure 1 for participant flow details.

### Well-being

#### Care partners

Well-being outcomes are shown in Table 3. Care partners were less likely to indicate social isolation concerns on the Caregiver Strain Instrument subscale at 3-months [χ^2^(4,34) = 13.96, *p* = .007] and 6-months [χ^2^(4,34) = 14.74, *p* = .005] compared to baseline. ID110 (care partner; dyadic enrollee) described how CFM™-D helped them to feel less isolated: “[It] made you realize you’re not alone and [to] reach out to people when you need help.” Mixed effect analysis showed that the gender and physical functioning of the retiring driver significantly influenced care partner isolation scores (see Supplementary Table 4). Caring for female retiring drivers was associated with higher isolation scores [β = 0.13, *t*(79) = −2.23]. Interestingly, care partners caring for retiring drivers with higher activities of daily living (ADL) scores, or more difficulty with functional activities, had lower isolation scores as measured by the Caregiver Strain Instrument [β = −1.16, *t*(79) = 2.92].

**Table 3.** Well-Being Measures over Time

Measure	Time point			Time point comparison									
	Baseline	3-months	6-months	Baseline vs 3-months follow-up					Baseline vs 6-months follow-up				
				*t*	*df*	*p* Value	Mean diff.	95% CI (LL, UL)	*t*	*df*	*p* Value	Mean diff.	95% CI (LL, UL)
Care partner													
*n*	34	27	26										
CSI mastery sum	3.8 (3.6)	4.0 (3.3)	4.2 (3.4)	1.06	23	.302	0.4	(−0.4, 1.2)	0.21	21	.833	0.1	(−0.8, 1.0)
CSI mastery elevated (>8), *n* (%)	3 (8.8)	3 (8.8)	3 (8.8)	6.91	4	<.001			9.93	4	.042		
Missing^a^, *n* (%)	7 (20.6)	8 (23.5)	10 (29.4)			—							
CSI relationship strain sum	6.1 (4.7)	4.3 (3.3)	5.7 (4.0)	1.83	25	.078	−1.5	(−0.2, 3.1)	0.93	24	.359	0.7	(−0.8, 2.2)
CSI relationship strain elevated (>10), *n* (%)	8 (23.5)	1 (2.9)	2 (5.9)	3.85	4	.141			6.81	4	.042		
Missing^a^, *n* (%)	1 (2.9)	8 (23.5)	9 (26.5)			—							
CSI health sum	6.3 (4.5)	5.7 (3.4)	5.5 (3.5)	0.26	25	.798	0.2	(−1.3, 1.7)	1.74	23	.096	0.8	(−0.2, 1.8)
CSI health elevated (>10), *n* (%)	7 (20.6)	1 (2.9)	1 (2.9)	3.85	4	.426			6.81	4	.146		
Missing^a^, *n* (%)	1 (2.9)	8 (23.5)	10 (29.4)			—							
CSI isolation sum	6.4 (1.9)	5.8 (2.2)	6.0 (1.8)	2.41	21	.025	0.8	(0.1, 1.5)	1.23	22	.231	0.3	(−0.2, 0.8)
CSI isolation elevated (>5), *n* (%)	17 (50.0)	10 (29.4)	10 (29.4)	13.96	4	.007			14.74	4	.005		
Missing^a^, *n* (%)	2 (5.9)	11 (32.4)	10 (29.4)			—							
DeJong loneliness sum	1.9 (1.6)	1.9 (1.6)	1.9 (1.6)	0.27	25	.788	−0.1	(−0.5, 0.7)	0.19	23	.852	0.0	(−0.4, 0.4)
Relationship closeness sum	19.5 (4.3)	19.7 (3.8)	18.5 (4.2)	0.00	25	1.000	0.0	(−0.9, 0.9)	1.31	24	.203	−0.8	(−0.4, 2.1)
Community engagement and service use sum	1.9 (1.9)	1.9 (1.6)	1.2 (1.6)	−0.93	26	.363	−0.2	(−0.6, 0.2)	0.77	25	.448	0.3	(−0.4, 1.0)
*Retired driver*													
Combined dyad and individual enrollment, *n*	32	29	29										
Geriatric depression sum	4.5 (3.9)	3.3 (2.7)	2.7 (2.4)	1.62	23	.118	0.8	(−0.2, 1.9)	2.56	24	.017	1.5	(0.3, 2.7)
DeJong loneliness sum	2.4 (2.2)	1.6 (1.8)	1.6 (1.7)	1.70	27	.101	0.6	(−0.1, 1.4)	1.60	27	.121	0.6	(−0.2, 1.4)
Individual enrolled only, *n*	16	14	15										
Relationship closeness sum	21 (1.8)	20.3 (2.3)	21.2 (1.9)	0.75	11	.470	0.5	(−1.0, 2.3)	−0.43	11	.678	0.3	(−2.1, 1.4)
Community engagement and service use sum	2.1 (1.6)	2.1 (1.6)	2.0 (1.9)	0.00	13	1.000	0.4	(−0.9, 0.9)	0.12	14	.903	0.1	(−1.1, 1.2)

*Notes*: CI = confidence interval; CSI = Caregiver Strain Index; *df* = degrees of freedom; diff. = difference; LL = lower limit; *t* = *t*-stasticic; UL = upper limit.

Data is presented as mean (standard deviation) unless otherwise specified.

^a^CSI scores missing if any response missing.

Additionally, care partners were less likely to report high relationship strain scores at 6-months compared to baseline [χ^2^(4,34) = 6.81, *p* = .042]. Dyad status and relationship closeness played a significant role in relationship strain. Care partners enrolled as a dyad and who had closer relationships with their retiring driver had less relationship strain. However, because dyad status and relationship closeness were highly correlated (*R*^2^ = −0.42, *p* ≤ .001), when both are in the model, only one is significant because of this multicollinearity [Supplementary Table 4 shows relationship closeness, β = −0.49, *t*(84) = −3.97; Voss, 2005]. ID290 (care partner; dyadic enrollee) expressed how the intervention positively affected their spousal relationship, indicating it helped them alleviate a sense of blame and feelings of being “less than” or ashamed for not driving. Another care partner (ID260; dyadic enrollee) shared an additional benefit of participating in CFM™-D with her spouse: “It created an atmosphere where talking about stopping driving was less emotional. We were able to talk about deciding when and what limitations should be placed on his driving.” No changes were observed for health and mastery subscales or other care partner well-being measures.

#### Retiring drivers

The retiring drivers showed no difference in well-being measures at baseline by enrollment status. They collectively indicated significantly fewer depressive symptoms at 6-months [*t*(24) = 2.56, *p* = .017]. Mixed effect analyses showed this decrease in depressive symptoms even controlling for covariates (see Supplementary Table 4). Qualitatively, retiring drivers shared how CFM™-D positively influenced their emotional health. ID211 (retiring driver; individual enrollee) noted: “Just opening up to someone who understands that there is a sense of loss and grief involved in no longer driving, is enormously helpful,” while another retiring driver (ID471; individual enrollee) shared: “It was successful for me because it helped ‘re-adjust’ my thinking regarding ‘poor me’ syndrome!”

Still, statistically, depressive symptoms were increased if the retiring driver was adjusting to retirement [i.e., had stopped driving; β = 1.53, *t*(83) = 2.25] and were lower on average with higher educational attainment [Bachelor or more; β = −2.83, *t*(83) = −2.55]. When evaluating retiring drivers who enrolled as part of a dyad, a linear regression model was the best fit. An increase in relationship closeness was significantly associated with decreases in depressive symptoms [β = −0.51, *t*(40) = −3.05], and adjusting to retirement was a negative influence [β = 1.39, *t*(40) = 2.16]. There were no statistically significant changes in relationship closeness, community engagement or service, or DeJong Loneliness Scale scores over time. That said, retiring drivers and care partners alike did share that talking with their coach provided a social outlet. As ID231 (retiring driver; individual enrollee) expressed: “I felt like I was talking to a friend, and when you live alone that’s so nice.”

### Driving Retirement Preparedness

At baseline, 21 retiring drivers (42%) had stopped driving and about 60% (17/29) of those still driving explicitly reported considering driving retirement. Preparedness measures were not statistically different by enrollment status at baseline. However, dyad retiring drivers were more likely to have spoken with a doctor or other healthcare provider about driving retirement, and individual retiring drivers were more likely to have had a traffic violation in the past 3 years.

#### Care partners

Descriptive changes in driving preparedness and behaviors are shown in Tables 4 and 5. Assessment for Readiness for Mobility Transition (ARMT) scores demonstrated significant improvement for care partners at the 3- and 6-month follow-up surveys [*t*(25) = 2.47, *p* = .021; *t*(23) = 2.10, *p* = .047, respectively]. However, care partners did not report improved mobility confidence (e.g., staying social and managing retirement conflicts). Even so, random slope models indicated that dyads enrolled had higher levels of readiness and mobility confidence, which they maintained over the study: Dyad fixed effects for both ARMT [β = −5.87, *t*(84) = −3.38] and mobility confidence [β = 12.2, *t*(84) = 3.95] indicate care partners enrolling as a dyad had better driving retirement preparedness from baseline through follow-up. Greater memory impairment was associated with less preparedness in the ARMT [β = 0.33, *t*(84) = 2.66], and higher ADL scores (more difficulty with activities) were associated with less mobility confidence [β = −0.67, *t*(84) = −2.75].

**Table 4.** Driving Retirement Phase and Activities over Time

Driving phase and activities	Time points					Time point comparison					
	Baseline	3-months	6-months	3-months cumulative	6-months cumulative	Baseline vs 3-months cumulative			Baseline vs 6-months cumulative		
Care partner, *n*	34	27	26			*t*	*df*	*p* Value	*t*	*df*	*p* Value
Stopped driving, *n* (%)	15 (44.1)	14 (41.2)	15 (44.1)	18 (52.9)	20 (58.8)	23.86	1	<.001	18.79	1	<.001
Missing, *n* (%)	0 (0)	7 (20.6)	8 (23.5)								
If still driving, considering driving retirement, *n* (%)^a^	9 (47.4)	6 (17.7)	5 (14.7)	11 (32.4)	12 (35.3)	56.25	4	<.001	51.85	4	<.001
Missing, *n* (%)	15 (44.1)	22 (64.7)	23 (65.7)								
Driving retirement activities, *n* (%):											
Driving evaluation	6 (17.7)	3 (8.8)	1 (2.9)	7 (20.6)	7 (20.6)	28.10	1	<.001	28.10	1	<.001
Talking with family or other support	10 (29.4)	11 (32.4)	5 (14.7)	17 (50.0)	18 (52.9)	14.17	1	<.001	12.59	1	<.001
Talking to their doctor or other healthcare provider	11 (32.4)	10 (29.4)	7 (20.6)	15 (44.1)	16 (47.1)	20.60	1	<.001	18.29	1	<.001
Limiting their driving (e.g., daytime and local roads)	16 (47.1)	16 (47.1)	9 (26.5)	23 (67.7)	23 (67.7)	14.45	1	<.001	14.45	1	<.001
Missing, *n* (%)	0 (0)	7 (20.6)	8 (23.5)								
Retiring driver, *n*	30	27	28								
Stopped driving, *n* (%)	12 (36.4)	9 (27.3)	11 (33.3)	14 (42.2)	17(51.5)	29.11	2	<.001	20.19	2	<.001
Missing, *n* (%)	1 (3.0)	7 (21.1)	6 (18.2)								
If still driving, considering driving retirement, *n* (%)^a^	12 (36.4)	13 (39.4)	12 (36.4)	19 (57.6)	21 (63.4)	34.10	2	<.001	27.65	4	<.001
Missing, *n* (%)	13 (39.4)	16 (48.5)	17 (51.5)								
Driving retirement activities, *n* (%):											
Driving evaluation	4 (12.1)	3 (9.1)	2 (6.1)	7 (21.2)	8 (24.2)	16.97	4	<.001	14.33	2	.001
Talking with family or other support	15 (45.5)	17 (51.5)	11 (33.3)	23 (69.7)	24 (72.7)	13.50	2	.001	11.65	2	.003
Talking to my doctor or other healthcare provider	7 (21.9)	6 (18.2)	6 (18.2)	11 (33.3)	14 (42.4)	17.88	2	<.001	14.33	2	.001
Limiting my driving (e.g., daytime and local roads)	16 (48.5)	14 (42.4)	14 (42.4)	20 (60.6)	20 (60.6)	20.43	2	<.001	20.43	2	<.001

*Notes*: *df* = degrees of freedom; *t* = *t*-statistic.

^a^Presented only if retiring driver was still driving.

**Table 5.** Driving Retirement Preparedness over Time

Driving retirement preparedness	Time points			Time point comparison									
	Baseline	3-months	6-months	Baseline vs 3-months follow-up					Baseline vs 6-months follow-up				
				*t*	*df*	*p* Value	Mean diff.	95% CI (LL, UL)	*t*	*df*	*p* Value	Mean diff.	95% CI (LL, UL)
Care partner, *n*	34	27	26										
Driving safety questionnaire sum^a^^,^^b^	28.4 (5.4)	29.8 (4.7)	29.5 (7.0)	−2.01	11	.070	−3.3	(−6.8, 0.3)	−1.58	9	.148	−3.1	(−7.5, 1.3)
Assessing readiness for mobility transitions sum^c^	26.5 (6.5)	23.7 (7.0)	24.2 (6.9)	2.47	25	.021	1.9	(0.3, 3.5)	2.10	23	.047	1.8	(0.0, 3.6)
Mobility confidence questionnaire sum^d^	42.8 (13.6)	47.6 (13.4)	42.4 (13.6)	−1.18	25	.249	−2.9	(−8.0, 2.2)	1.08	24	.291	1.6	(−1.5, 4.8)
Retiring driver, *n*	32	29	29										
Driving and safety questionnaire sum^a^^,^^b^	23.4 (6.1)	25.5 (3.7)	24.3 (5.7)	−0.97	13	.35	−1.5	(−4.8, 1.8)	−0.97	12	−.261	−0.5	(−4.3, 3.4)
Assessing readiness for mobility transitions sum^c^	25.6 (6.1)	21.7 (6.5)	22.9 (6.4)	3.25	24	.003	3.7	(1.4, 6.1)	2.66	24	.014	3.0	(0.7, 5.4)
Mobility confidence questionnaire sum^d^	56.9 (24.0)	70.9 (15.7)	65.6 (20.7)	−3.82	24	<.001	12.6	(−19.3, −5.8)	−1.82	25	.080	−7.7	(−16.3, 1.0)

*Notes*: CI = confidence interval; *df* = degrees of freedom; diff. = difference; LL = lower limit; *t* = *t*-stasticic; UL = upper limit.

^a^Presented only if retiring driver was still driving.

^b^Higher scores = less safe.

^c^Higher scores = less prepared.

^d^Range = 0–70 for the care partner and 0–90 for the retiring driver.

ID70 (care partner; dyadic enrollee) provided an overview of how the intervention influenced their readiness for driving retirement, including learning about alternative transportation: “Realized how easy [the Uber rideshare app] was. [Retiring driver] began realizing the importance of limiting his driving considering his health.” Another care partner (ID242; individual enrollee) described that CFM™-D provided “language and tactics and things to use to start to talk to [her dad, retiring driver] and the confidence to be able to do that.” ID110 (care partner; dyadic enrollee) cited the tailored transportation information as being particularly helpful in building their mobility confidence: “[The coach] looked up all the local transportation options for us too, so we have all that when we need it.”

#### Retiring drivers

ARMT scores significantly improved at the 3- and 6-month follow-ups among retiring drivers [*t*(24) = 3.25, *p* = .003; *t*(24) = 2.66, *p* = .014, respectively]. In contrast, to care partner mobility confidence, retiring drivers had significantly improved mobility confidence scores at 3-month follow-up [*t*(24) = −3.82, *p* < .001], but this significance was not sustained at 6-months [*t*(25) = −1.82, *p* = .080]. ID391 (retiring driver; dyadic enrollee) relayed:

It provided us a sense of confidence that when the time comes … we’re not going to panic … Having the conversation [is] something we can move to and deal with and think about and probably do it sooner rather than later, rather than resist it forever.

A care partner (ID110; dyadic enrollee) of a retiring driver who had already retired from driving added that it buoyed the retiring driver’s confidence in his decision: “[He] realize[d] he’s not alone and kind of reaffirm[ed] the fact that he’s making the right decision by not driving and the different alternatives available.” Random intercept models provided the best fit for retiring drivers. Greater cognitive impairment was associated with less mobility confidence [β = −1.51, *t*(84) = −2.51]. For dyadic retiring drivers, higher relationship closeness scores were associated with better AMRT [β = −1.09, *t*(42) = −2.2] and mobility confidence scores [β = 4.31, *t*(42) = 2.84]. Nevertheless, mobility confidence had large variability (see variances in Supplementary Table 4) for both individual and dyadic retiring drivers.

Both care partners and retiring drivers engaged in more driving retirement behaviors and activities after baseline (e.g., talking with family about it), with the biggest increases in activity overlapping the intervention sessions in the first three months. ID160 (care partner; dyadic enrollee) described increased comfort with driving alternatives after joining CFM™-D, “We have called on friends more to help with transportation things when I’m not able to and that’s something we never would have asked before, but just sort of realizing that it’s necessary.” Reported driving safety concerns and driving restrictions (e.g., weather and highways) were not significantly different over time.

## Discussion

The long course of ADRD and the cognitive, behavioral, and functional challenges that often accompany disease progression result in several transitions that can complicate the life experience of the person with ADRD as well as those who care for them. Transition points during the course of dementia care can serve as important intervention targets, as these are often time periods that can cause particular emotional, social, or health upheaval for people with ADRD and their care partners (i.e., driving retirement). The objective of the current study was to determine if CFM™-D, a psychosocial and psychoeducational intervention, delivered before, during, or following driving retirement was perceived as beneficial by persons with ADRD and their care partners, and whether changes in key outcomes occurred when receiving the intervention.

Evaluation of CFM™-D treatment receipt data indicated that both individual and dyad participants judged the intervention’s delivery mode, structure, and content as highly useful. Almost all participants indicated they would recommend the intervention to someone else in a similar situation, and regardless of dyadic or individual care partner receipt, the intervention was deemed beneficial. One potentially complicating issue in this preliminary evaluation was the attrition that occurred among care partners, particularly those who participated in CFM™-D as individual enrollees. Although our empirical analysis did not identify differential attrition, the need to develop stronger engagement and retention strategies for these participants is necessary.

In terms of well-being, participation in CFM™-D appeared associated with reductions in elements of caregiver burden, as isolation and relationship strain declined over the 6-month follow-up period. One of the principal clinical processes of CFM™-D is to offer psychosocial support during the driving retirement transition. This coaching element of CFM™-D is critically missing from self-guided resources (Byszewski et al., 2017; Jouk & Tuokko, 2017) and decision-making tools (Carmody et al., 2015; Chang et al., 2021; Polzer et al., 2020). Regular, ongoing engagement with CFM™-D coaches may reduce perceptions of care partner strain related to isolation and may have also facilitated greater understanding, communication skills, and a sense of partnership between care partners and persons with dementia (i.e., the retiring drivers). In particular, CFM™-D appeared to have direct well-being benefits for retiring drivers themselves. Over the course of the intervention, retiring drivers reported significantly fewer depressive symptoms. As prior reviews of dementia care interventions emphasize, incorporating the person with ADRD into programmatic and clinical content is a successful element of intervention strategies (Gitlin & Hodgson, 2015; Gitlin et al., 2020). Moreover, tailoring such content to the needs of persons with ADRD and their care partners, which is an essential approach of CFM-D, appears to further enhance retiring drivers’ overall well-being and may help explain the tentative success of the intervention. The context of participation also appeared to influence the effects of CFM™-D on these mechanisms, as care partners who participated in a dyad (who often reported closer relationships) were more likely to indicate both improved readiness and confidence over time.

Among the key proposed mechanisms of action of CFM™-D is that the psychosocial and psychoeducational content of the intervention improves perceptions of readiness for and confidence in managing issues related to driving retirement. Findings suggest the CFM™-D intervention improved perceptions of readiness and confidence among participants and may explain why the intervention improved outcomes for care partners and retiring drivers. The ongoing connection with coaches, as ID160 (care partner; dyadic enrollee) described, “sort of hold[s] [the participant] accountable” in activity planning and goal-setting follow-through. This may be a vital ingredient to CFM™-D that sets it apart from other driving retirement interventions currently available. Although statistical power and design precluded formal mediational analysis to empirically establish this clinical process, the strong conceptual grounding of CFM™-D from prior work in Australia (Liddle et al., 2007, 2013; Scott et al., 2020b) and this preliminary evaluation highlights confidence and readiness as intervention mechanisms that may spur well-being benefits for care partners and people living with dementia throughout the driving retirement transition. One retiring driver (ID11; dyadic enrollee) summarized:

This experience has given me confidence to be proactive in pursuing new activities, making new connections, and come to accept that I am prepared to stop driving [whenever] that time comes, without stress [and with] a sense of confidence in making that decision without dread or fear of how I will get around.

As is noted in behavioral intervention science in general, intervention development that neither tests nor establishes a mechanism of change leads to an inefficient process of translation (2020, 2021; Onken, 2022). By not understanding how or why an intervention works, researchers and later adopters of a given intervention who are unaware of its essential ingredients may scale or adapt activities that fundamentally alter the intervention and its outcomes. The conceptual grounding and testing receipt of CFM™-D and its association with key mechanisms (confidence and readiness for driving retirement) inform future efficacy and, if successful, subsequent dissemination and implementation efforts of this promising intervention.

There were limitations. Although the design was adequate for a pilot evaluation of CFM™-D, there was not a control condition, so any inferences related to efficacy or effectiveness of the intervention are not possible. As noted earlier, a trend in attrition emerged, although attrition biases were not detected.

Even so, the findings from our evaluation of CFM™-D establish initial evidence to address driving retirement in ADRD populations, a key transition that, to date, has few evidence-based resources to guide families or healthcare providers, especially ones focused on the relationship complexities that can arise. This is not only a scientific gap, but a clinical one as optimal dementia care includes addressing driving issues and concerns of persons living with dementia and their care partners (Callahan et al., 2014; Odenheimer et al., 2014). We are at the early stages of testing the benefits and mechanisms of CFM™-D and more rigorous efficacy testing is needed to establish the intervention’s potential in the United States. Nonetheless, scalability of CFM™-D is a particularly important issue to consider even in the Stage I phase of evaluation (per the NIH Stage Model; Onken et al., 2014). In our current pilot, two doctoral-prepared interventionists delivered the content of the intervention. However, for CFM™-D to appeal to healthcare systems, community-based organizations, and other likely adopters, future evaluations of the intervention must incorporate implementation measures (e.g., organizational workflow alignment and sustainability potential; Lewis et al., 2017; Proctor, 2020) and explore intervention adaptations to achieve the broadest possible benefits for families struggling with driving retirement and dementia (Curran et al., 2012, 2022; Kirk et al., 2020; Wiltsey Stirman et al., 2019).

## Conclusion

This preliminary efficacy evaluation of CFM™-D for U.S. retiring drivers living with dementia and their care partners indicated a range of positive outcomes. The psychosocial and psychoeducational content of the intervention suggested mechanisms (readiness and confidence) that may explain why CFM™-D benefited care partners and retiring drivers. The specification of underlying mechanisms, as well as contextual moderators of benefit (e.g., participation as an individual or dyad), offered further insights regarding potential CFM™-D benefits. Future research on CFM™-D in the United States aims to test the intervention with a more rigorous, controlled design to further ascertain the benefits, as well as mechanisms, of CFM™-D.

## Supplementary Material

igae022_suppl_Supplementary_Figures_S1_Tables_S1-S4
