# Supplementary material for: CarFreeMe™-Dementia: Potential Benefits of a Driving Retirement Intervention Supporting Persons With Dementia and Their Families
Source: Innov Aging. 2024 Mar 1;8(3):igae022. doi: 10.1093/geroni/igae022 (PMC10962632; doi:10.1093/geroni/igae022)
Supplement: igae022_suppl_Supplementary_Figures_S1_Tables_S1-S4 [file igae022_suppl_supplementary_figures_s1_tables_s1-s4.docx]

*Innovation in Aging* Supplementary Material: Colleen M. Peterson, Stephanie Ingvalson, Robyn W. Birkeland, Katie W. Louwagie, Theresa L Scott, Nancy A. Pachana Jacki Liddle, Louise Gustafsson, & Joseph E. Gaugler. CarFreeMe™-Dementia: Potential Benefits of a Driving Retirement Intervention Supporting Persons with Dementia and their Families.

*Supplementary Figure 1:* *Participant Flow CONSORT Diagram*


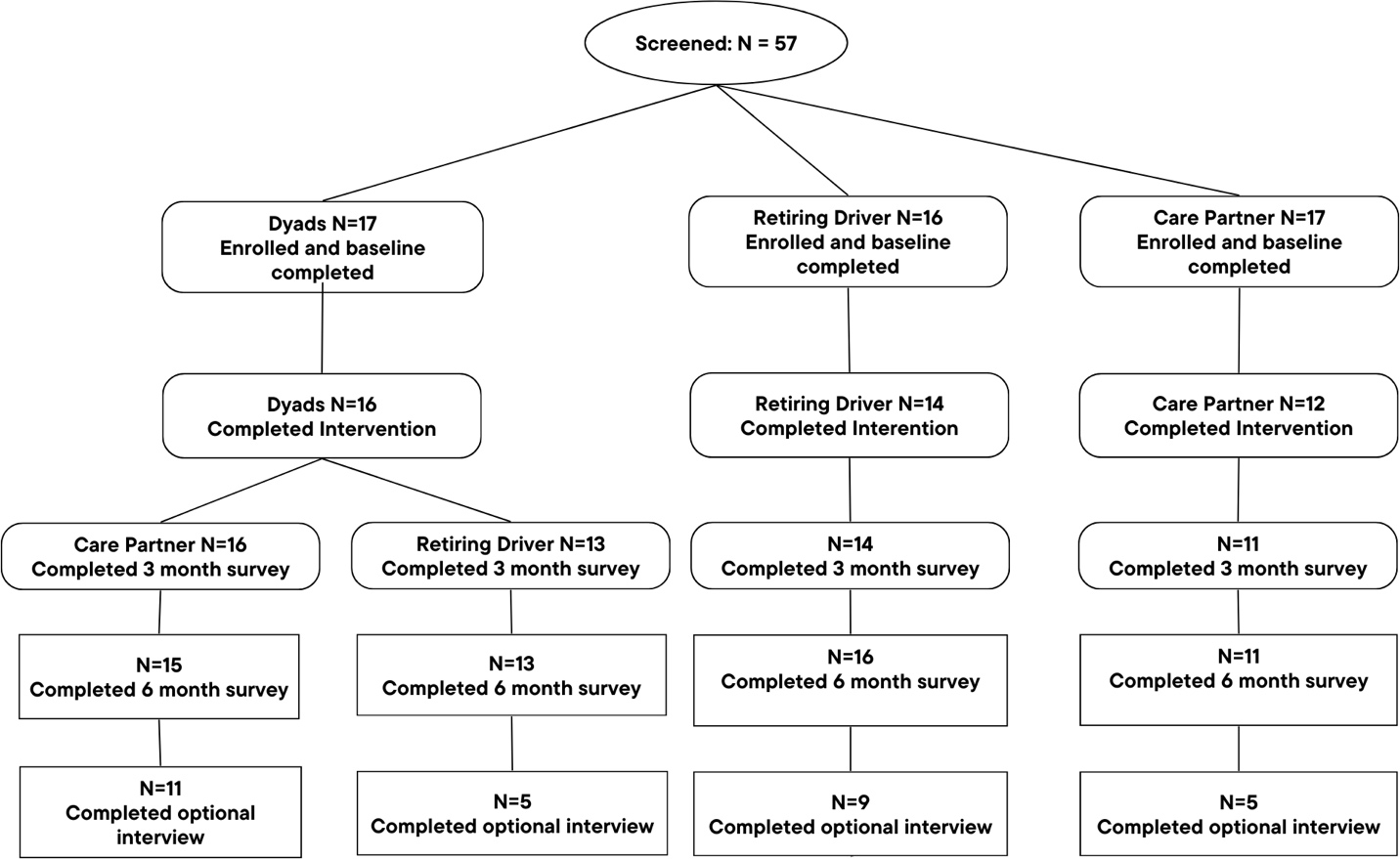


*Note*. Two of the care partners that enrolled were caring for the same person; one withdrew and the other, who had participated in all the coaching sessions, officially enrolled to replace their family member at a later point in the study.

*Supplementary Table 1: Summary of Assessments Administered*

| **Assessment Tool** | **Description** | **Range** | **Care Partner** | **Retiring Driver-Dyad** | **Retiring Driver-Single** |
| --- | --- | --- | --- | --- | --- |
| Demographics | Age, gender, ethnicity/race, married, income, education, children, work status | NA | x |  | x |
| Driving cessation phase and activities | Assessed phase and activities regarding driving cessation | NA | x | x | x |
| Caregiver Driving Safety Questionnaire (Iverson et al., 2010) | 14 items assessing history of crashes or traffic citations, including at-fault status, driving mileage, and driving practices | NA | x | x | x |
| Excursion Questionnaire (author-developed) | 5 items measuring the method and safety of transport used away from home | NA | x | x | x |
| Confidence managing driving cessation-Care Partner (Scott et al., 2019) | 7 items assessing confidence in managing various aspects of driving cessation | 0-70 | x |  |  |
| Confidence managing driving cessation-Retiring Driver (Scott et al., 2019) | 9 items assessing confidence in managing various aspects of driving cessation | 0-90 |  | x | x |
| Assessment for Readiness of Mobility Transition short form (Meuser & Berg-Weger 2012) | 8 items assessing readiness for driving retirement on emotional and attitudinal dimensions | 0-40 | x | x | x |
| Activities of Daily Living or Instrumental Activities of Daily Living (Modified) (Katz et al., 1963) | 12 items assessing help required with activities | 0-24 | x |  | x |
| Community-based services (Sonnega et al., 2013) | 19 items assessing community-based services use | 0-19 | x |  | x |
| Cognitive/Memory Impairment (Pearlin et al., 1990) | 8 items assessing the severity of memory loss, communication deficits, and recognition failures | 0-32 | x |  | x |
| Revised-Memory and Behavior Problems Checklist (Teri et al., 1992) | 24 items assessing dementia-related behavioral issues | 0-96 | x |  |  |
| Relationship Closeness Scale (Whitlatch, et al., 2001) | 6 items assessing relationship closeness between the care partner and retiring driver | 0-24 | x | x |  |
| Relationship Question | If needed, do you have someone you could turn to for help? (for example, a supportive family member) | 0-1 |  |  | x |
| DeJong Gierveld Loneliness Scale (6-item) (De Jong Gierveld & Van Tilburg, 2006; De Jong Gierveld & Van Tilburg, 2010) | 6 items assessing emotional and social loneliness | 0-6 | x | x | x |
| Geriatric Depression Scale short-form (Yesavage & Sheikh, 1986) | 15 items screening for depression in the elderly | 0-15 |  | x | x |
| Caregiver Strain Instrument (Benjamin Rose Institute Bass, Noelker & Reschlin, 1996) | 19 item screening for caregiver strain | 0-52 | x |  |  |
| - | Mastery subscale | 0-12 | x |  |  |
| - | Strain (isolation, health) subscale | 0-30 | x |  |  |
| - | Isolation subscale | 0-10 | x |  |  |
| CarFreeMe™-Dementia Intervention Receipt Checklist-Care Partner | 15-item on utility of the intervention content | NA | x |  |  |
| CarFreeMe™-Dementia Intervention Receipt Checklist-Retiring Driver | 8-item on utility of the intervention content | NA |  | x | x |

*Supplementary Table 2: Baseline Participant Characteristics by Dyadic Enrollment Status*

| **Care Partner (n=34)** | **Care Partner-Single (n=17)** | | **Care Partner-Dyad (n=17)** | |  |
| --- | --- | --- | --- | --- | --- |
|  | **n** | **%** | **n** | **%** | **p-value** |
| Female | 15 | 88.2 | 15 | 88.3 | 1.000 |
| Age, M(SD) | 64.4 | 14.3 | 68.9 | 8.8 | 0.269 |
| White | 17 | 100.0 | 15 | 88.2 | 0.145 |
| Married | 15 | 88.2 | 16 | 94.1 | 0.545 |
| Bachelor's degree or higher | 12 | 70.3 | 13 | 76.5 | 0.697 |
| Number of living children, M(SD) | 1.8 | 1.6 | 2.1 | 1.5 | 0.582 |
| Annual Income of US$80,000 or more^a^ | 9.0 | 52.9 | 7 | 46.7 | 0.723 |
| Spouse of care recipient | 9 | 52.9 | 16 | 94.1 | **0.007** |
| Employed | 4 | 23.5 | 2 | 11.8 | 0.368 |
| Lives with caregiver^b^ | 10 | 62.5 | 16 | 94.1 | **0.026** |
| **Care Partner Well-being** |  |  |  |  |  |
| CSI mastery sum | 3.7 | 4.4 | 3.9 | 3.1 | 0.919 |
| CSI relationship strain sum | 7.9 | 5.2 | 4.3 | 3.6 | **0.025** |
| CSI health sum | 6.8 | 4.4 | 5.8 | 4.5 | 0.508 |
| CSI isolation sum | 6.6 | 1.9 | 6.3 | 1.9 | 0.711 |
| DeJong loneliness sum | 1.9 | 1.9 | 1.9 | 1.5 | 0.910 |
| Relationship closeness sum | 17.4 | 5.0 | 21.5 | 2.3 | **0.005** |
| **Retiring Driver Dementia Characteristics (Care Partner reported)** |  |  |  |  |  |
| Years recognizing memory concerns | 4.1 | 2.6 | 4.5 | 4.4 | 0.468 |
| Limitations to activities of daily living (ADL) sum | 8.6 | 4.8 | 7.8 | 5.2 | 0.625 |
| Memory impairment sum | 11.0 | 5.2 | 6.4 | 3.5 | **0.006** |
| Diagnosed with AD/ADRD, n(%) | 12.0 | 70.6 | 11.0 | 64.7 | 0.714 |
| **Retiring Driver driving behaviors (Care Partner reported)** | **n** | **%** | **n** | **%** |  |
| Stopped driving, n(%) | 9 | 52.9 | 6 | 35.3 | 0.300 |
| If still driving, considering driving retirement, n(%)^b^ | 4 | 50.0 | 5 | 45.5 | 0.845 |
| **In last 3 years any…** |  |  |  |  |  |
| Traffic violation | 2 | 12.5 | 1 | 6.3 | 0.544 |
| Caused or in accident | 4 | 25.0 | 3 | 18.8 | 0.669 |
| Accidents at fault | 3 | 18.8 | 2 | 13.3 | 0.682 |
| **Weekly miles driven** |  |  |  |  |  |
| 0 | 0 | 0.0 | 1 | 9.1 | 0.509 |
| 1-25 | 5 | 71.4 | 6 | 54.6 |  |
| 26-50 | 2 | 28.6 | 2 | 18.2 |  |
| 51-100 | - | - | - | - |  |
| 100+ | 0 | 0.0 | 2 | 18.2 |  |

*Supplementary Table 2: Baseline Participant Characteristics by Dyadic Enrollment Status Continued*

| **Retiring Driver (n=32)** | **Retiring Driver-Single (n=16)** | | **Retiring Driver-Dyad (n=16)** | |  |
| --- | --- | --- | --- | --- | --- |
|  | **n** | **%** | **n** | **%** | **p-value** |
| Female | 11 | 68.8 | 5 | 31.2 | **0.034** |
| Age, M(SD) | 72.5 | 6.7 | 73.9 | 5.5 | 0.530 |
| White | 12 | 75.0 | 16 | 100.0 | **0.033** |
| Married | 6 | 37.5 | 15 | 93.8 | **0.001** |
| Bachelor's degree or higher | 9 | 56.3 | 12 | 75.0 | 0.264 |
| Number of living children, M(SD) | 1.86 | 1.5 | 2.125 | 1.4 | 0.625 |
| Annual Income of US$30,000 or more ^a^ | 12 | 75 | 12 | 80.0 | 0.739 |
| Lives with caregiver | 4 | 25.0 | 15 | 93.8 | **0.000** |
| **Dementia Characteristics** |  |  |  |  |  |
| Years recognizing memory concerns | 6.4 | 7.3 | 4.4 | 4.5 | 0.410 |
| Limitations to activities of daily living (ADL) sum | 2.3 | 2.6 | 7.8 | 5.3 | **0.001** |
| Memory impairment sum | 12.4 | 2.8 | 6.3 | 3.6 | **0.000** |
| Diagnosed with AD/ADRD, n(%)^c^ | 2.0 | 12.5 | 9.0 | 56.3 | **0.009** |
| **Retiring driver driving behaviors** | **n** | **%** | **n** | **%** |  |
| **In last 3 years any…** |  |  |  |  |  |
| Traffic violation | 4 | 26.7 | 0 | 0.0 | **0.027** |
| Caused or in accident | 4 | 26.7 | 2 | 12.5 | 0.318 |
| Accidents at fault | 4 | 26.7 | 2 | 12.5 | 0.318 |
| **Weekly miles driven** |  |  |  |  |  |
| 0 | 6 | 40.0 | 6 | 37.5 | 0.729 |
| 1-25 | 5 | 33.3 | 7 | 43.8 |  |
| 26-50 | 3 | 20.0 | 3 | 18.8 |  |
| 51-100 | 1 | 6.7 | 0 | 0.0 |  |
| 100+ | - | - | - | - |  |

Notes: CSI=Caregiver Strain Index;

^a^ Median reported income by retiring driver/care partner

^b^ Only asked if reported the retiring driver was still driving

^c^ Does not include mild cognitive impairment (MCI)

*Supplementary Table 3: CarFreeMe™-Dementia Phase II Intervention Receipt*

| **Items** | **3- month** | |  | **6-month** | |  |
| --- | --- | --- | --- | --- | --- | --- |
| **Care Partner - CarFreeMe helped me….** | **M(SD)** | **% Agree** | **NA** | **M(SD)** | **% Agree** | **NA** |
| 1. Learn the importance of planning early for driving retirement | 4.5(0.7) | 86.4 | 5 | 4.4(0.7) | 90.9 | 3 |
| 2. Explore important aspects of planning early | 4.6(0.7) | 90.9 | 5 | 4.4(0.6) | 95.7 | 1 |
| 3. Increase knowledge of or use of safe alternative modes of transportation such as bus, light rail, taxi | 4.4(0.8) | 84.0 | 2 | 4.2(0.8) | 81.0 | 4 |
| 4. Learn ways to continue or increase participation in the community | 4.2(0.8) | 76.9 | 1 | 4.0(0.7) | 78.3 | 2 |
| 5. Develop strategies to help manage mobility | 4.2(0.8) | 80.0 | 2 | 4.0(0.8) | 75.0 | 1 |
| 6. Better understand changes that can take place with dementia such as brain changes, behavior, thinking | 4.4(0.8) | 85.2 | 0 | 4.4(0.6) | 96.0 | 0 |
| 7. Better understand the skills required for driving and how dementia affects those skills | 4.3(0.9) | 83.3 | 3 | 4.2(0.6) | 90.5 | 3 |
| 8. Become aware of coping strategies for adjusting to life without driving | 4.5(0.6) | 92.3 | 1 | 4.2(0.7) | 83.3 | 1 |
| 9. Learn about or express concerns and feel validated related to grief/loss of the experience of driving | 4.2(0.8) | 76.9 | 1 | 4.2(0.7) | 87.0 | 2 |
| 10. Be supported through an emotional adjustment to the loss of driving (autonomy, independence, etc.) | 4.3(0.8) | 80.0 | 2 | 4.1(0.8) | 70.0 | 3 |
| 11. Explore others’ experiences with retiring from driving | 3.9(0.7) | 72.0 | 2 | 3.9(0.7) | 72.7 | 3 |
| 12. Learn how to set goals | 4.2(0.9) | 72.0 | 2 | 4.0(0.9) | 61.9 | 3 |
| 13. Learn ways to conserve energy | 3.8(0.9) | 63.6 | 4 | 3.8(0.9) | 52.4 | 3 |
| 14. Overall: I would recommend CarFreeMe to others in a similar situation as my relative is | 4.7(0.6) | 96.3 | 0 | 4.5(0.7) | 92.0 | 0 |
| 15. Overall: I would recommend CarFreeMe to others in a similar situation as I am | 4.5(0.9) | 96.0 | 0 | 4.6(0.7) | 91.7 | 1 |
|  | **3-month** | |  | **6-month** | |  |
| **Retiring Driver-combined - CarFreeMe helped me….** | **M(SD)** | **% Agree** | **NA** | **M(SD)** | **% Agree** | **NA** |
| 1. Plan for when I will not drive anymore | 4.3(0.8) | 82.6 | 0 | 4.2(0.8) | 86.4 | 5 |
| 2. Find other ways to get around such as the bus, light rail, taxi, rides from friends or family, etc. | 4.4(0.8) | 92.0 | 0 | 4.2(0.7) | 84.0 | 3 |
| 3. Continue to participate in my community | 4.1(0.9) | 76.9 | 0 | 4.2(0.6) | 88.9 | 1 |
| 4. Learn more about dementia and changes that can happen in my brain and in my day-to-day life | 4.4(0.7) | 96.3 | 0 | 4.3(0.7) | 85.7 | 0 |
| 5. Express my feelings about not driving anymore (such as grief, relief, etc.) | 4.5(0.6) | 96.2 | 0 | 4.1(0.9) | 80.8 | 2 |
| 6. Be supported through this experience of not driving anymore | 4.4(0.6) | 96.0 | 0 | 4.2(0.9) | 85.2 | 1 |
| 7. Hear stories about others who had retired from driving and how they adjusted | 4.0(1.0) | 84.6 | 0 | 3.7(1.1) | 66.7 | 1 |
| 8. Overall: I would recommend CarFreeMe to others in a similar situation as I am | 4.6(0.6) | 96.3 | 0 | 4.5(.9) | 92.6 | 1 |

Note: M(SD)=mean(standard deviation); NA=not applicable % Agree= agree or strongly agree and does not include the participants responding NA or did not select any answer; One care partner’s scores were removed from the six-month CFM™-D review due to the strong likelihood the responses were in error as they were completely incongruent with both their previous review on surveys and personal interactions. The scores were also extreme outliers. Even so, sensitivity analyses indicate removal of their scores result in approximately 1% difference in mean scores.

**Supplementary Table 4.** *Linear Mixed Effect Models of Primary Outcomes Sum Scores*

| Effect | Primary Outcome | | | | | | | | | | | | |
| --- | --- | --- | --- | --- | --- | --- | --- | --- | --- | --- | --- | --- | --- |
| Care Partner | Isolation | | | | Relationship Strain | | | ARMT-Short Form^a^ | | | Mobility Confidence | | |
| Random Effects | Variance | SD | |  | Variance | SD |  | Variance | SD |  | Variance | SD |  |
| Dyad intercept | - | - | |  | - | - |  | 12.59 | 3.80 |  | 26.93 | 12.09 |  |
| Intercept | 1.52 | 0.57 | |  | 6.55 | 2.36 |  | - | - |  | - | - |  |
| Residual | 1.30 | 0.28 | |  | 5.61 | 1.14 |  | 7.38 | 1.50 |  | 58.93 | 11.83 |  |
| Fixed effects | Estimate | SE | | 95% CI  (LL, UL) | Estimate | SE | 95% CI  (LL, UL) | Estimate | SE | 95% CI  (LL, UL) | Estimate | SE | 95% CI  (LL, UL) |
| Intercept | 5.87 | 0.51 | | (4.90.00, 6.90) | 16.27 | 2.30 | (11.67, 20.88) | 26.39 | 1.74 | (22.92, 29.86) | 27.82 | 6.48 | (14.85, 40.79) |
| 3-month survey | -0.96 | 0.34 | | (-1.64, -0.28) | -1.44 | 0.64 | (-2.73, -0.15) | -2.19 | 0.75 | (-3.70, -0.68) | 4.37 | 2.08 | (0.21, 8.53) |
| 6-month survey | -0.59 | 0.33 | | (-1.25, 0.08) | -0.94 | 0.66 | (-2.27, 0.39) | -2.39 | 0.81 | (-4.00, -0.77) | 0.59 | 2.15 | (-3.71, 4.90) |
| Dyad (yes) | - | - | | - | -1.30 | 1.16 | (-3.65, 1.05) | -5.87 | 1.74 | (-9.40, -2.34) | 12.18 | 3.08 | (5.91, 18.44) |
| Retiring driver Female | 0.13 | 0.04 | | (0.04, 0.21) | - | - | - | - | - | - | - | - | - |
| RCS sum | - | - | | - | -0.49 | 0.12 | (-0.73, -0.24) | - | - | - | 0.72 | 0.33 | (0.06, 1.39) |
| MI sum | - | - | | - | - | - | - | 0.33 | 0.13 | (0.08, 0.58) | - | - | - |
| ADL sum | -1.16 | 0.52 | | (-2.22, -0.10) | - | - | - | - | - | - | -0.68 | 0.25 | (-1.17, -0.18) |
| Retiring Driver | Depressive Symptoms | | | |  |  |  | ARMT-Short Form | | | Mobility Confidence | | |
| Random Effects | Variance | SD | |  | - | - | - | Variance | SD |  | Variance | SD |  |
| Intercept | -2.83 | 1.11 | |  | - | - | - | 25.47 | 7.94 |  | 285.22 | 96.00 |  |
| Residual | 1.53 | 0.68 | |  | - | - | - | 15.78 | 3.11 |  | 151.08 | 30.87 |  |
| Fixed effects | Estimate | SE | | 95% CI  (LL, UL) | - | - | - | Estimate | SE | 95% CI  (LL, UL) | Estimate | SE | 95% CI  (LL, UL) |
| Intercept | 5.83 | 0.95 | | (3.90, 7.75) | - | - | - | 25.87 | 1.14 | (23.59, 28.16) | 71.47 | 6.84 | (0.00, 57.8) |
| 3-month survey | -0.94 | 0.47 | | (-1.88, -0.01) | - | - | - | -2.66 | 1.09 | (-4.84, -0.48) | 12.29 | 3.39 | (0.00, 5.49) |
| 6-month survey | -1.78 | 0.46 | | (-2.7, -0.85) | - | - | - | -2.99 | 1.09 | (-5.17, -0.81) | 8.98 | 3.37 | (0.01, 2.22) |
| Stopped driving | 1.53 | 0.68 | | (0.18, 2.89) | - | - | - | - | - | - | - | - | - |
| Bachelor’s or more | -2.83 | 1.11 | | (-5.10, -0.56) | - | - | - | - | - | - | - | - | - |
| MI sum | - | - | | - | - | - | - | - | - | - | -1.51 | 0.60 | (0.02, -2.71) |
| Retiring Driver (Dyad only) | Depressive Symptoms^b^ | | | |  |  |  | ARMT-Short Form | | | Mobility Confidence | | |
| Random Effects | Variance | SD |  | | - | - | - | Variance | SD |  | Variance | SD |  |
| Intercept | - | - |  | | - | - | - | 16.60 | 8.91 |  | 285.22 | 96.00 |  |
| Residual | - | - |  | | - | - | - | 20.51 | 5.89 |  | 151.08 | 30.87 |  |
| Fixed effects | Estimate | SE | 95% CI  (LL, UL) | | - | - | - | Estimate | SE | 95% CI  (LL, UL) | Estimate | SE | 95% CI  (LL, UL) |
| Intercept | 14.00 | 3.58 | (6.74, 0.42) | | - | - | - | 48.80 | 10.46 | (27.61, 70) | -31.00 | 32.19 | (-96.21, 34.2) |
| 3-month survey | -1.13 | 0.77 | (-2.69, 0.67) | | - | - | - | -3.14 | 1.80 | (-6.83, 0.56) | 13.26 | 5.51 | (1.93, 24.6) |
| 6-month survey | -0.90 | 0.77 | (-2.47, 2.7) | | - | - | - | -2.21 | 1.78 | (-5.87, 1.44) | 6.13 | 5.45 | (-5.08, 17.35) |
| Stopped driving | 1.39 | 0.64 | (0.09, -0.17) | | - | - | - | - | - | - | - | - | - |
| RCS sum | -0.51 | 0.17 | (-0.85, 21.26) | | - | - | - | -1.09 | 0.49 | (-2.09, -0.09) | 4.31 | 1.52 | (1.23, 7.39) |

*Note*. SD=Standard deviation; SE=Standard error; RCS=Relationship closeness score; MI=Memory impairment.

^a^ ARMT=Assessment for Readiness of Mobility Transition (higher scores = less prepared).

^b^ Dyad depressive symptoms were a regression (not random effects) model

*Supplementary Text: Semi-Structured Interview Guides*

**Family Care Partner**

**Semi-Structured Interview Prompts [Adaptations for Conversation]**

**Introduction** *Thank you so much for participating in the CarFreeMe program. I would like to ask some questions about your experience and record our conversation for data analysis.*

1. To get us started, where is your relative in the process of driving retirement?

2. What was your overall impression of the CarFreeMe program?

3. What did you get out of the CarFreeMe program?

- Did your relative benefit from your involvement in the program? If so, how?
- *If relative was part of sessions: Did your relative benefit from their involvement in CarFreeMe? If so, how?*

4. What did you feel was not helpful or was missing from the program?

5. How did CarFreeMe affect your relative’s readiness for driving retirement? [*If retired: How did CarFreeMe help your relative adjust to driving retirement?]*

6. How did CarFreeMe help *you* get ready for your relative’s driving retirement? [*If retired: How did CarFreeMe help you adjust to your relative’s driving retirement?]*

- How did CarFreeMe affect your stress and well-being?
- Did it impact your ability to manage your relative’s driving retirement? If yes, how so?

7. Did CarFreeMe help your relative to feel involved in their driving decisions? *[i.e., Everyday driving decisions (grocery, friends) or driving retirement]*

8. Did the program make it easier to talk about driving issues? *[i.e., With your relative, family, or care professionals?]*

9. Did your relative’s driving or use of transportation change because of CarFreeMe? How? *[i.e., alternative transportation, getting rides from family and friends]*

- Do you think CarFreeMe helped your relative get where they wanted to go?

10. Did CarFreeMe help your relative to stay involved in activities, social engagements, and/or in their community?

11. After completing the CarFreeMe program, what, if anything, do you feel like you need now?

- Do you have any remaining concerns about driving or transportation for your relative and yourself? [If yes, “Tell me more.”]

12. Thank you for helping us learn more about your experiences with CarFreeMe. Is there anything else you would like to add?

**Retiring Driver**

**Semi-Structured Interview Prompts [Adaptations for Conversation]**

**Introduction** *Thank you so much for participating in the CarFreeMe program. I would like to ask some questions about your experience and record our conversation for data analysis.*

1. To get us started, are you still driving?

2. What was your overall impression of the CarFreeMe program?

3. What did you get out of the CarFreeMe program?

4. What did you feel was not helpful or was missing from the program?

5. Did CarFreeMe affect how you felt about retiring from driving? If yes, how so?

[*If retired: How did CarFreeMe help you adjust to driving retirement?]*

- Did CarFreeMe affect how ready you felt about retiring from driving? If yes, how so?
- How did CarFreeMe affect your stress and well-being?

7. Did CarFreeMe help you to feel involved in your driving decisions? *[i.e., Everyday driving decisions (grocery, friends) or driving retirement]*

8. Did the program make it easier to talk about driving issues? *[With your family, friends, or care professionals?]*

9. Did your driving or what you use for transportation change because of CarFreeMe? How? *[i.e., alternative transportation, getting rides from family and friends]*

- Do you think CarFreeMe helped you get where you wanted to go?

10. Did CarFreeMe help you stay involved in activities, social engagements, and/or in your community?

11. After completing the CarFreeMe program, what do you feel like you need now, if anything?

- Do you have any concerns about driving or transportation? [If yes, “Tell me more.”]

12. Thank you for helping us learn more about your experiences with CarFreeMe. Is there anything else you would like to add?
